# Factors Associated With Neck Hematoma After Thyroidectomy: A Retrospective Analysis Using a Japanese Inpatient Database

**DOI:** 10.1097/MD.0000000000002812

**Published:** 2016-02-18

**Authors:** Sayaka Suzuki, Hideo Yasunaga, Hiroki Matsui, Kiyohide Fushimi, Yuki Saito, Tatsuya Yamasoba

**Affiliations:** From the Department of Otolaryngology, Head and Neck Surgery, Faculty of Medicine (SS, YS, TY) and Department of Clinical Epidemiology and Health Economics, School of Public Health (HY, HM), University of Tokyo; and Department of Health Policy and Informatics, Tokyo Medical and Dental University Graduate School of Medicine (KF), Tokyo, Japan.

## Abstract

To identify risk factors for post-thyroidectomy hematoma requiring airway intervention or surgery (“wound hematoma”) and determine post-thyroidectomy time to intervention.

Post-thyroidectomy hematoma is rare but potentially lethal. Information on wound hematoma in a nationwide clinical setting is scarce.

Using the Japanese Diagnosis Procedure Combination database, we extracted data from records of patients undergoing thyroidectomy from July 2010 to March 2014. Patients with clinical stage IV cancer or those with bilateral neck dissection were excluded because they could have undergone planned tracheotomy on the day of thyroidectomy. We assessed the association between background characteristics and wound hematoma ≤2 days post-thyroidectomy, using multivariable logistic regression analysis.

Among 51,968 patients from 880 hospitals, wound hematoma occurred in 920 (1.8%) ≤2 days post-thyroidectomy and in 203 (0.4%) ≥3 days post-thyroidectomy (in-hospital mortality = 0.05%). Factors significantly associated with wound hematoma ≤2 days post-thyroidectomy were male sex (odds ratio [OR] 1.52, 95% confidence interval [CI] 1.30–1.77); higher age (OR 1.01, 95% CI 1.00–1.02); overweight or obese (OR 1.22, 95% CI 1.04–1.44); type of surgery (partial thyroidectomy for benign tumor compared with: total thyroidectomy, benign tumor [OR 1.95, 95% CI 1.45–2.63]; partial thyroidectomy, malignant tumor [OR 1.21, 95% CI 1.00–1.46]; total thyroidectomy, malignant tumor [OR 2.49, 95% CI 1.82–3.49]; and thyroidectomy for Graves disease [OR 3.88, 95% CI 2.59–5.82]); neck dissection (OR, 1.53, 95% CI 1.05–2.23); antithrombotic agents (OR 1.58, 95% CI 1.15–2.17); and blood transfusion (OR 5.33, 95% CI 2.39–11.91).

Closer monitoring of airway and neck is recommended for patients with risk factors, and further cautious monitoring beyond 3 days post-thyroidectomy.

## INTRODUCTION

Thyroidectomy is generally safe and commonly performed for benign or malignant tumors and Graves disease and in the United States, short-stay thyroidectomy on an outpatient basis is increasingly common.^1–3^ A potentially devastating early complication after thyroidectomy is the formation of a neck hematoma resulting in airway obstruction. Acute airway distress such as this deserves special attention because it is unpredictable and potentially lethal unless promptly evaluated and relieved emergently with surgical procedures. However, concern remains regarding the time interval from initial thyroidectomy to the onset of hematoma in light of the safety of short-stay thyroidectomy.

According to recent studies with large sample sizes (n ≥ 1000), the incidence of postsurgical neck hematoma ranges from 0.30% (42/13,817)^4^ to 1.7% (519/30,142).^5^ Also, several reports indicate that hematoma formation can occur more than 24 h after initial thyroidectomy: 10% (7/70) (n = 6830)^6^; 17% (1/6) (n = 837)^7^; 17% (36/207)^8^; 19% (8/42) (n = 13,817)^4^; and 28.5% (2/7) (n = 504).^9^

A recent study using the US Nationwide Inpatient Sample revealed that male sex, inflammatory thyroid conditions, partial thyroidectomy, chronic renal disease, and bleeding disorders were predictors of hematoma formation after inpatient thyroidectomy.^1^ However, the results were limited because the database included only inpatient data and lacked information on airway intervention or readmission for hematoma after discharge following short hospital stay.

The present study used a Japanese national inpatient database to investigate adverse events after thyroidectomy. Unlike the United States, most patients undergoing thyroidectomy remain in hospital for several days after surgery in Japan, which enabled us to conduct a relatively long-term observational study of patients after thyroidectomy.

## METHODS

The study protocol was approved by the institutional review board of the University of Tokyo. Because the data were anonymous, the requirement for informed consent was waived.

### Data Source

The Diagnosis Procedure Combination (DPC) database, a Japanese nationwide inpatient database, contains administrative claims data and discharge abstracts. For each patient, the DPC includes the main diagnosis, comorbidities at admission, and complications after admission, coded using the corresponding International Classification of Diseases (ICD)-10 codes; surgical interventions and medical procedures, with the original Japanese codes; patient characteristics (age, sex, weight, height, Brinkman index); drugs and devices used during hospitalization; type of hospital (academic or nonacademic); admission status (with or without disturbance of consciousness); and discharge status. The database also includes information on the dates for each drug administered or procedure performed.

All 82 academic hospitals across Japan are obliged to participate in the DPC database, while community hospitals participate voluntarily. The number of included patients in 2012 was over 6.8 million from approximately 1000 hospitals, representing more than half of all inpatient admissions to acute care hospitals across Japan. The data collection period was 6 months (July 1 to December 31) in 2010 and was extended to the entire year (12 months) beginning in 2011. The codes corresponding to each surgery, clinical procedures, and medication are almost complete because they are compulsory items for reimbursement of healthcare costs. The responsible physicians are required to record the information on each patient with reference to each patient's medical chart to maximize the accuracy of these data. The details of the database are described elsewhere.^10^

### Patient Selection

Data for patients undergoing thyroidectomy between July 1, 2010 and March 31, 2014 were extracted from the database. We excluded patients with the following conditions: tracheotomy performed before thyroidectomy; intubated before thyroidectomy; surgery for other diseases during hospitalization; disturbance of consciousness on admission; and chemotherapy during hospitalization. We also excluded patients with bilateral neck dissection or those with clinical stage IV thyroid cancer because these patients may have received planned tracheotomy on the day of thyroidectomy.

### Patient Characteristics

The following patient characteristics were assessed: age; sex; body mass index (BMI, kg/m^2^); smoking status (smoker [current or former smoker] or never smoker); type of thyroidectomy (partial thyroidectomy for benign tumor, total thyroidectomy for benign tumor, partial thyroidectomy for malignant tumor, total thyroidectomy for malignant tumor, and total (near-total) thyroidectomy for Graves disease); blood transfusion; use of antithrombotic agents during hospitalization; and type of hospital (academic or nonacademic). Comorbidities on admission were also assessed, using the recorded ICD-10 codes as well as Japanese text data: diabetes mellitus (ICD-10 codes: E10–E14); hypertension (I10); ischemic cardiac diseases (I20–I25); chronic obstructive pulmonary diseases (J43, J44); renal failure (N18, N19); hepatic disorders (K70–K77); cerebrovascular diseases (I60–I69); hyperlipidemia (E78x); and bleeding disorders (D66, D67, D680, D682). Antithrombotic agents included antiplatelet agents (aspirin, cilostazol, ticlopidine, clopidogrel, sarpogrelate, beraprost, and icosapentate) and anticoagulant agents (warfarin, dabigatran, edoxaban, rivaroxaban, and apixaban). We calculated hospital volume (HV) using the annual number of thyroid surgeries performed in each hospital during the study period. We classified the patients into low (≤29), medium (30–74), and high (≥75) HV groups. According to the World Health Organization definitions, we classified BMI as follows: underweight (<18.50 kg/m^2^); normal weight (18.50–24.99 kg/m^2^); or overweight and obese (≥25.00 kg/m^2^).^11^ We further divided normal weight into low-normal (18.50–22.99 kg/m^2^) or high-normal (23.00–24.99 kg/m^2^).

### Outcome Measurements

The primary outcome was wound hematoma requiring tracheotomy, intubation, or surgical removal during the initial hospitalization or at readmission. The interval (days) between thyroidectomy and these procedures was calculated.

### Statistical Analysis

The incidence proportions of wound hematoma requiring tracheotomy, intubation, or surgical removal were compared between the subgroups using the χ^2^ test or Fisher exact test. To evaluate the relationships between background characteristics and the occurrence of wound hematoma requiring tracheotomy, intubation, or surgical removal, we performed a multivariable logistic regression analysis with adjustment for within-hospital clustering using a generalized estimating equation. Variance inflation factors for independent variables were calculated to assess multicollinearity between the independent variables. A variance inflation factor >10 was considered to show multicollinearity. A 2-sided *P* value <0.05 was considered significant. The Statistical Package for Social Sciences version 20.0 (IBM SPSS Corp., Armonk, NY) was used for all statistical analyses.

## RESULTS

A total of 57,115 patients who underwent thyroidectomy from July 1, 2010 to March 31, 2014 were identified. Of these, 5148 patients were excluded because they received tracheotomy before thyroidectomy (n = 33); were intubated before thyroidectomy (n = 48); received surgeries for other diseases during hospitalization (n = 2114); had disturbance of consciousness on admission (n = 142); received chemotherapy during hospitalization (n = 304); underwent bilateral neck dissection (n = 1035); or had clinical stage IV thyroid cancer (n = 2290). Finally, 51,967 eligible patients from 880 hospitals were included.

**FIGURE 1:**
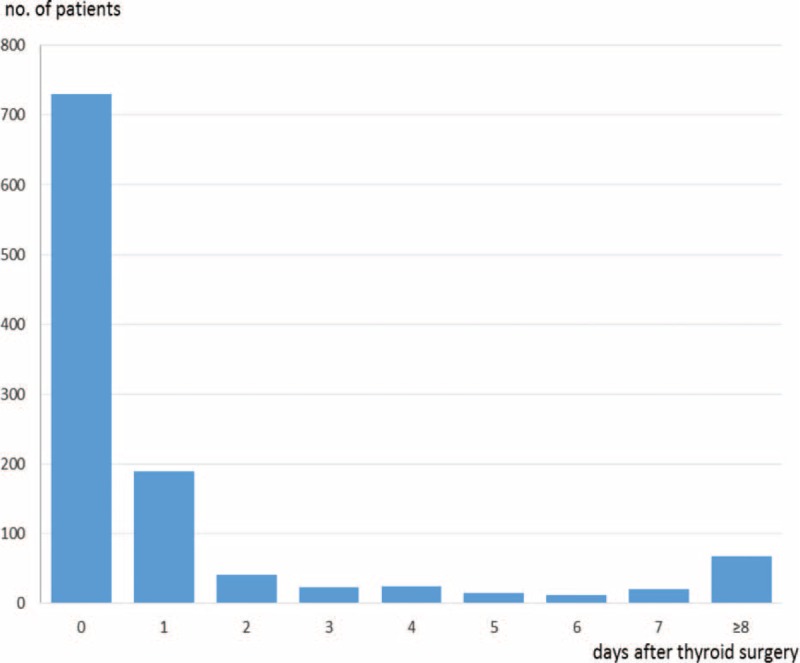
Interval from thyroidectomy to airway intervention including surgery for hematoma.

A total of 1123 (2.2%) patients had wound hematoma requiring tracheotomy, intubation, or surgical removal. Of these patients, 920 (1.8% of all) had wound hematoma within 2 days (day 0 and day 1) post-thyroidectomy, while 203 had wound hematoma ≥3 days post-thyroidectomy (Figure 1).

During the study period, a total of 468 patients received tracheotomy (tracheotomy combined with intubation in 29 patients; tracheotomy with surgery for hematoma in 47 patients; tracheotomy alone in 395 patients), 179 patients received intubation (intubation combined with surgery for hematoma in 15 patients; intubation alone in 138 patients), and 564 patients received surgery for hematoma (surgery for hematoma alone in 505 patients). Of the 920 patients with wound hematoma within 2 days of thyroidectomy, 397 patients received tracheotomy (tracheotomy combined with intubation in 4 patients; tracheotomy with surgery for hematoma in 22 patients), 160 patients received intubation (intubation combined with surgery for hematoma in 11 patients), and 399 patients received surgery for hematoma. Among the 203 patients with wound hematoma at ≥3 days post-thyroidectomy, 71 patients received tracheotomy (tracheotomy combined with intubation in 25 patients; tracheotomy with surgery for hematoma in 25 patients), 19 patients received intubation (intubation combined with surgery for hematoma in 4 patients), and 165 patients received surgery for hematoma. Three patients received all 3 interventions.

The median (interquartile range) of post-thyroidectomy length of stay was 7 days (5–9 days) in all patients, 11 days (8–21 days) in patients with wound hematoma, and 7 days (5–9 days) in those without wound hematoma.

Table 1 summarizes the background characteristics and the numbers of patients with wound hematoma requiring tracheotomy, intubation or surgical removal. The mean ± standard deviation age was 54.8 ± 15.7 years and the male:female ratio was 1:3. More than 1-quarter of the patients were overweight or obese and more than two-thirds did not have a smoking habit. Partial thyroidectomy for benign tumor was performed in 32.1% of the patients, followed by partial thyroidectomy for malignant tumor in 30.1% and total thyroidectomy for malignant tumor in 27.1%.

**TABLE 1:**
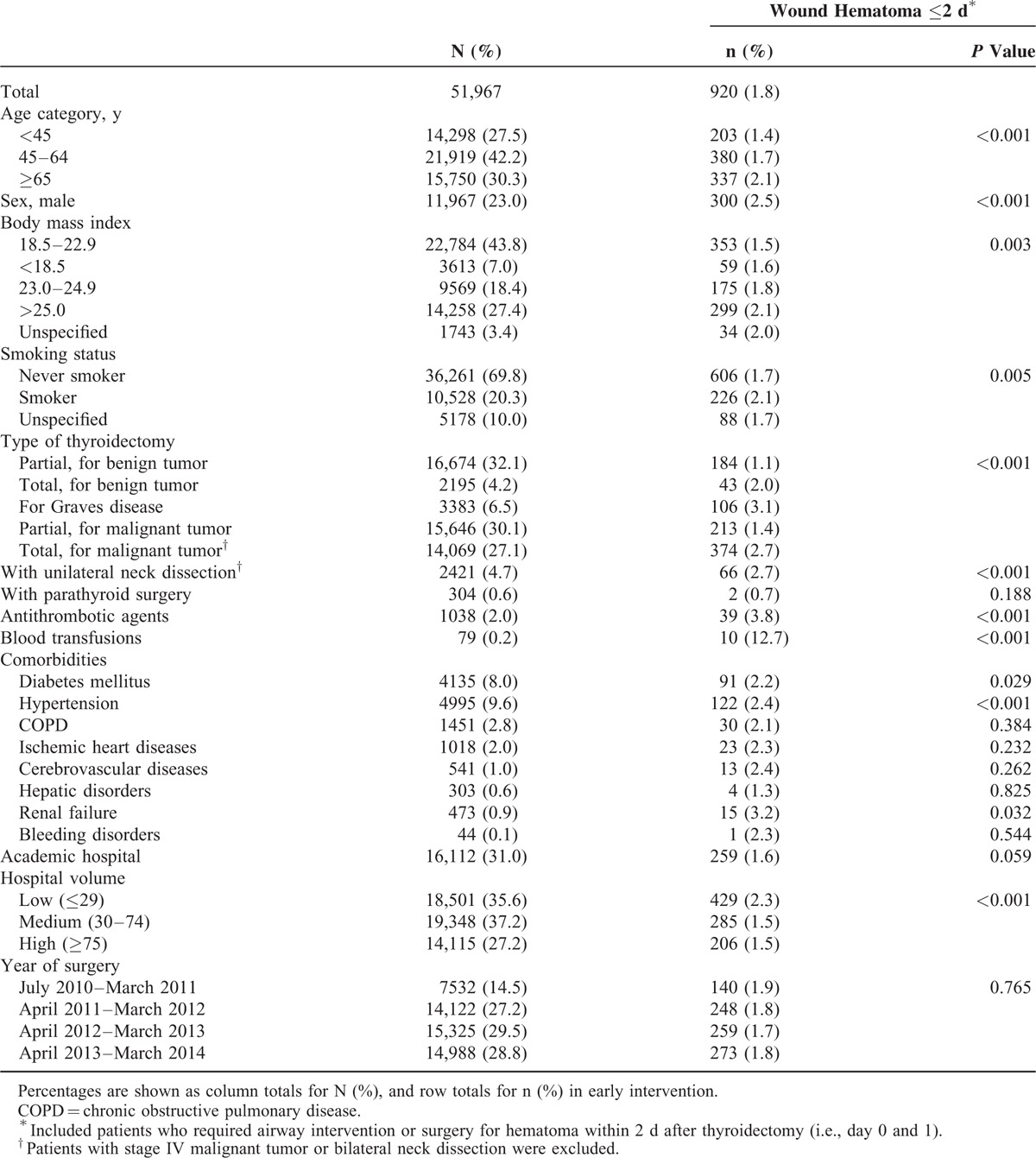
Background Characteristics and Incidence of Wound Hematoma ≤2 d After Thyroidectomy^∗^

Among all patients, 4.7% underwent unilateral neck dissection, 8.0% had diabetes mellitus, 9.6% had hypertension, 2.0% received antithrombotic agents during hospitalization, and 31.0% underwent surgery in academic hospitals. HV was distributed with a median of 42 thyroid surgeries per fiscal year (interquartile range, 22–79). Two hospitals had more than 1500 thyroidectomies per year, 1 hospital had approximately 200 thyroidectomies per year, and the remaining had <200.

Table 2 shows the results of multivariable logistic regression analysis. Variance inflation factors for all independent variables were <1.5, indicating no multicollinearity between the variables. Higher incidence proportions of wound hematoma were significantly associated with higher age, male sex, BMI ≥ 25.0, surgery for Graves disease and total thyroidectomy for either benign or malignant tumor, neck dissection, antithrombotic agents, and blood transfusion on the day of thyroidectomy. Smoking status, comorbidities, type of hospital, or HV were unassociated with the occurrence of wound hematoma.

**TABLE 2:**
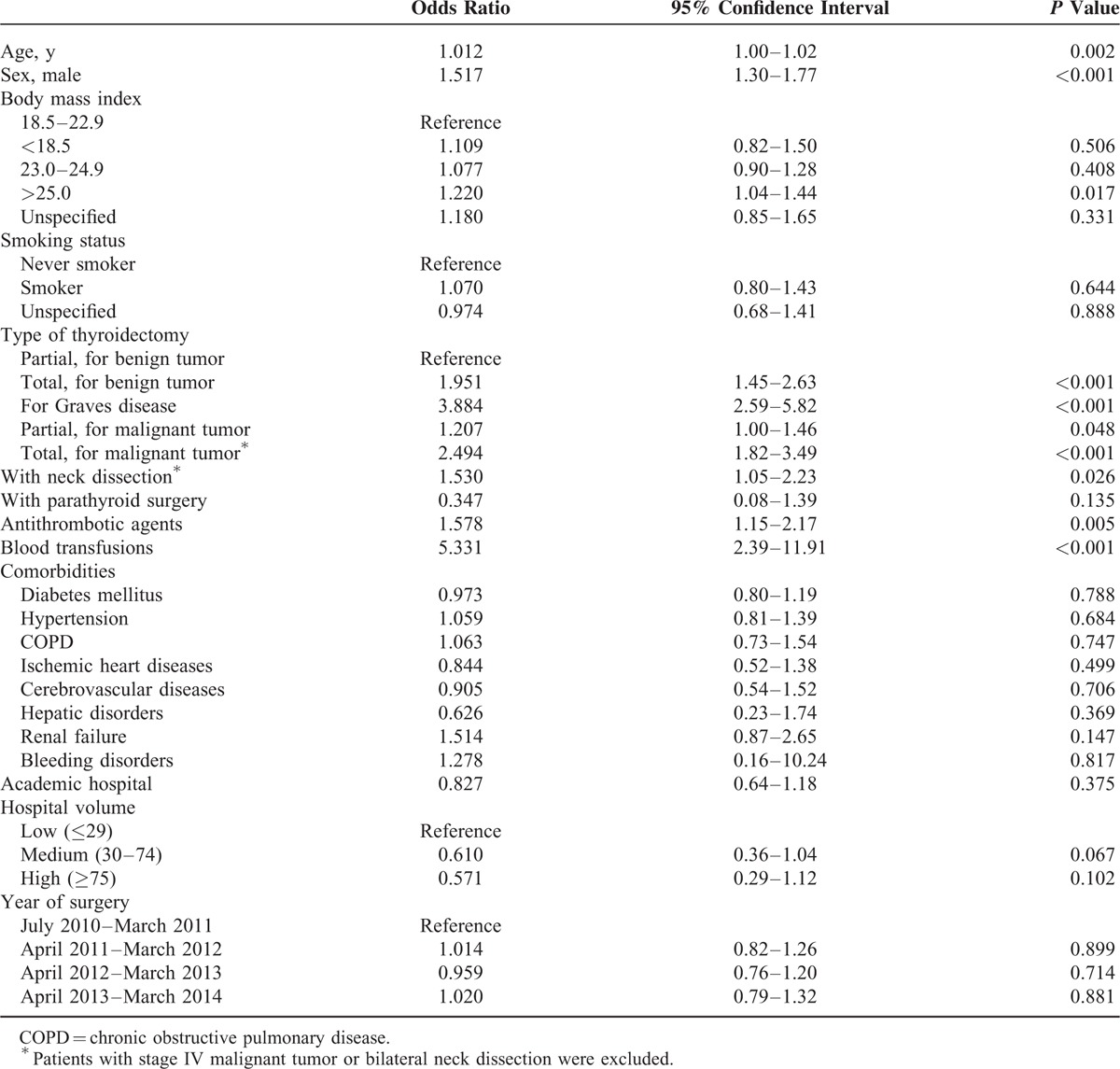
Logistic Regression Analysis for Airway Intervention Including Surgery for Hematoma Within 2 d of Thyroidectomy

Twenty-two patients (0.05%) died during hospitalization, including 1.0% (9/920) of patients with hematoma requiring treatment within 2 days and 0.03% (13/51,047) of those without hematoma (Fisher's exact test, *P* < 0.001). Eight patients died within 7 days post-thyroidectomy, consisting of 3 of 9 patients with hematoma requiring treatment within 2 days and 5 of 13 patients without hematoma.

## DISCUSSION

Using a Japanese nationwide inpatient database, we investigated the incidence proportions and risk factors for wound hematoma requiring tracheotomy, intubation, or surgical removal in patients undergoing thyroidectomy. Among 51,967 eligible patients, 1123 patients had wound hematoma requiring tracheotomy, intubation, or surgical removal during the same hospitalization or at readmission. Of these, 920 (82%) had wound hematoma within 2 days of thyroidectomy. To our knowledge, this is the first study to investigate the factors influencing wound hematoma after thyroidectomy, and to illustrate the time from thyroidectomy to the occurrence of wound hematoma, in a nationwide clinical setting.

In this study, patients were hospitalized and observed postoperatively for a median period of 7 days, which is consistent with previous findings in Japan.^12^ We believe that this period is long enough to capture a delayed occurrence of wound hematoma.

It is of note that nearly 20% of wound hematomas occurred ≥3 days post-thyroidectomy. Most previous studies reported only early-phase wound hematomas that developed within 24 h following thyroidectomy, with few studies discussing longer times.^4,7,9,13,14^ Our results indicated that clinicians should be aware of the probability of late-phase wound hematoma occurring ≥3 days post-thyroidectomy.

A recent large-scale study in the United States focused exclusively on surgery for hematoma.^1^ Airway intervention is also important as an outcome measure for evaluating post-thyroidectomy airway complications. When severe hematoma with rapid mucosal edema and upper airway swelling is detected, tracheotomy or intubation would generally be the first-choice emergency treatment, rather than surgery. In fact, the present study showed that only 399 of 920 patients with wound hematoma received surgery for the hematoma.

Our study revealed an association between higher age and the occurrence of wound hematoma, as in a previous study.^15^ Unlike the results in a recent US study,^1^ the present study showed obesity and overweight patients were at higher risk of developing wound hematoma requiring interventions than were normal or underweight patients. We speculate that when postoperative hematoma or edema occurred in the upper airway, overweight patients were more vulnerable to airway emergency than were normal weight patients, possibly because of reduced airway capacity. Smoking status was not associated with wound hematoma requiring early intervention, as in a recent US study.^1^

Previous studies revealed that surgery for large lesions,^5,8,16^ bilateral thyroidectomy,^5^ inflammatory thyroid conditions,^1^ and surgery for Graves disease^8,15^ had a negative impact on postsurgical hematoma formation. Our study identified that surgery for Graves disease, total thyroidectomy (benign or malignant), thyroidectomy for malignant tumor, and thyroidectomy with neck dissection were independent risk factors for wound hematoma requiring early intervention. These results are biologically plausible for 2 reasons. First, compared with unilateral or partial thyroidectomy, total thyroidectomy usually leaves a large dead space after surgery. Thyroidectomy with neck dissection requires more complex procedures and a wider surgical area. These conditions may promote postsurgical exudation and hematoma formation. Second, thyroid parenchyma in patients with Graves disease is known to have increased vascularity, which is considered a risk factor for postprocedural bleeding. According to a previous study, known causes of post-thyroidectomy hematoma include slipping of ligatures on major vessels, reopening of cauterized veins, retching and vomiting, Valsalva maneuver, increased blood pressure, and continuous exudation from the original thyroid location, in the postoperative period.^17^

Only a few studies have evaluated the effect of antithrombotic agents on hematoma formation and these showed inconsistent results.^1,6,8,9^ Our study revealed that patients who received antithrombotic agents during hospitalization were significantly more likely to suffer wound hematoma, suggesting the need for even more meticulous hemostatic procedures during thyroidectomy.

The present study revealed no association between institutional factors (academic hospital, HV, or year of surgery) and the occurrence of wound hematoma. In Japan, thyroidectomy is widely performed by endocrine surgeons, general surgeons, and otolaryngologists in both academic and nonacademic hospitals.

Several limitations should be acknowledged in the present study. First, this was a retrospective cohort study without randomization of treatment assignment (type of surgery, with or without neck dissection). Second, history of surgery in the neck region,^7,16,18^ preoperative inflammatory conditions in Graves disease, tumor location or size, methods of hemostasis (cold dissection or electrocautery), use of suction drainage or neck compression after thyroidectomy, surgeon's experience,^19^ wound hematoma without any intervention, and severity of hematoma, which were not available in the database, might act as unrecorded confounding factors. Third, generally in administrative claims databases, the records for comorbidities are less accurate than in prospective studies.

## CONCLUSIONS

Among thyroidectomy patients (with benign tumor, Graves disease, or clinical stage I–III malignancy; without bilateral neck dissection), 1.8% received unplanned airway intervention or surgery for hematoma within 2 days after thyroidectomy, in this study. The development of airway emergency or hematoma requiring surgical procedure was significantly associated with male sex, higher age, overweight or obese, Graves disease, antithrombotic agents, total thyroidectomy, malignant tumor, neck dissection, and blood transfusion on the day of thyroidectomy. Of all interventions, approximately 20% were performed after the third post-thyroidectomy day, suggesting the need for continuous attention to the airway and observation for wound complications.
